# An intelligent network framework for driver distraction monitoring based on RES-SE-CNN

**DOI:** 10.1038/s41598-025-91293-5

**Published:** 2025-02-26

**Authors:** Jichong Lei, Zining Ni, Zhiqiang Peng, Hong Hu, Jun Hong, Xiaoyong Fang, Cannan Yi, Changan Ren, Muhammad Abdul Wasaye

**Affiliations:** 1 School of Safety and Management Engineering, Hunan Institute of Technology, Hengyang, Hunan China; 2 School of Electrical and Information Engineering, Hunan Institute of Technology, Hengyang, Hunan China; 3 School of Computer Science and Engineering, Hunan Institute of Technology, Hengyang, Hunan China

**Keywords:** Human-machine co-driving, Distracted driving recognition, Object detection, Deep Learning, Computer Vision, Mechanical engineering, Computer science

## Abstract

As the quantity of motor vehicles and drivers experiences a continuous upsurge, the road driving environment has grown progressively more complex. This complexity has led to a concomitant increase in the probability of traffic accidents. Ample research has demonstrated that distracted driving constitutes a primary human - related factor precipitating these accidents. Therefore, the real - time monitoring and issuance of warnings regarding distracted driving behaviors are of paramount significance. In this research, an intelligent driver state monitoring methodology founded on the RES - SE - CNN model architecture is proposed. When compared with three classical models, namely VGG19, DenseNet121, and ResNet50, the experimental outcomes indicate that the RES - SE - CNN model exhibits remarkable performance in the detection of driver distraction. Specifically, it attains a correct recognition rate of 97.28%. The RES - SE - CNN network architecture model is characterized by lower memory occupancy, rendering it more amenable to deployment on vehicle mobile terminals. This study validates the potential application of the intelligent driver distraction monitoring model, which is based on transfer learning, within the actual driving environment.

## Introduction

With the continuous improvement of economic levels, the global number of motor vehicles has been increasing exponentially. By 2020, the total number of drivers in China had reached 450 million, with around 370 million motor vehicles^1^. However, road infrastructure has clearly not kept pace with the demand for vehicles, leading to increasingly severe traffic problems; at the same time, in-vehicle information systems (such as media players and navigation devices) have introduced more distractions, raising the risk of traffic accidents. The *2020 Global Status Report*indicated that approximately 1.35 million people die from road traffic accidents globally each year, with as many as 50 million people injured, comparable to the death rates from hepatitis and HIV. A significant portion of road traffic accidents is attributed to distracted driving, with the number of such accidents and the associated economic losses steadily increasing^2,3^.

Distracted driving is defined as any activity that diverts the driver from the task of safe driving. Currently, there are two main types of methods for identifying distracted driving^4^: one involves monitoring vehicle state information to reflect the driver’s driving condition, and the other involves using physiological and visual sensors to assess the driver’s state. In 2018, Tang et al.^5^ were the first to use vehicle driving information to establish a driving performance detection model based on support vector machines (SVM) to detect distracted states and develop a driving performance evaluation system. Lin et al.^6^ used smartphone sensors to collect vehicle driving data to monitor distracted driving behavior. Wang et al.^7^ modeled distracted states using brainwave signals and simple mathematical calculations through SVM during the driving process. In 2010, Yang et al.^8^ first used electrocardiogram (ECG) morphology from different body postures to determine driving posture and assess the driving state, proposing the concept of using physiological indicators to monitor driver status. Yin et al.^9^ combined the ResNet neural network with pose estimation to establish a model for recognizing distracted driving behavior. Liu et al.^10^ used the MTCNN network model for facial detection and judged the driver’s distracted state by obtaining eye gaze direction and head posture. Although substantial research on driver distraction monitoring has been conducted both domestically and internationally, the following issues remain: (1) The data collection methods based on driving data are costly and constrained by many factors, with poor practicality; (2) The physiological data collection methods significantly impact driving behavior, and the device costs are difficult to accept.

To address these issues, this paper establishes a driver distraction recognition model based on RES-SE-CNN neural network architectures. Compare to three classical model VGG19, DenseNet121 and ResNet50, this model enables the rapid and accurate identification of driver distraction, playing a crucial role in advancing driver behavior monitoring technology and providing strong support for achieving intelligent traffic management.

## Theoretical principles

### Classical model introduction

VGG19, DenseNet121 and ResNet50 are three representative deep convolutional neural networks (CNNs) that have had a significant impact on the field of computer vision. Each model has a unique design philosophy and application scenario, and they are widely used in tasks such as image classification and object detection, achieving excellent results in various competitions^11^.

VGG19, proposed by the Visual Geometry Group at Oxford University, became well-known for its outstanding performance in the ImageNet Large Scale Visual Recognition Challenge (ILSVRC)^12^. The design idea of VGG19 is to stack multiple small 3 × 3 convolutional kernels to increase the network’s depth. This structure is simple yet significantly enhances feature extraction capabilities. Specifically, VGG19 contains 16 convolutional layers and 3 fully connected layers, uses ReLU as the activation function, and employs max-pooling layers to reduce the size of feature maps. DenseNet121 is a version of the Dense Convolutional Network (DenseNet)^13^. The core idea of DenseNet is dense connections, where each layer receives the feature maps from all previous layers as input. This maximizes feature reuse and alleviates the vanishing gradient problem. DenseNet121 consists of several dense blocks and transition layers, with each block containing varying numbers of convolutional layers, all connected to previous layers. DenseNet121 has shown exceptional performance on multiple image classification tasks, especially on the ImageNet dataset, demonstrating its excellent feature reuse ability and training stability. ResNet50 is a type of Residual Network (ResNet)^14^. The key innovation of ResNet is the introduction of residual blocks, which address the degradation problem in deep networks through skip connections. ResNet50 comprises 50 convolutional layers, divided into multiple residual blocks, with each block containing several convolutional layers and skip connections. ResNet50 won the ILSVRC in its early years, proving its great potential in deep learning. Its residual learning framework has become an important reference for subsequent deep learning model designs. The advantages and disadvantages of the three neural networks are summarized in Table 1.

**Table 1:** Comparison of advantages and disadvantages of each model.

Model	Advantages	Disadvantages
VGG19	Simple structure, easy to understand and implement; improves performance by increasing network depth	Large number of parameters, high computational and storage demands; longer training time
DenseNet121	Efficient feature utilization, reduces parameter count; alleviates gradient vanishing, aids network training	High memory demands due to dense connections; complex structure, increasing implementation and debugging difficulty
ResNet50	Enables training of very deep networks without degradation; residual connections improve gradient flow and training effectiveness	Complex structure with more hyperparameters to tune compared to traditional CNNs; still has high computational overhead for specific tasks

### RES-SE-CNN model architecture

#### Convolutional neural network (CNN)

**Fig. 1:**
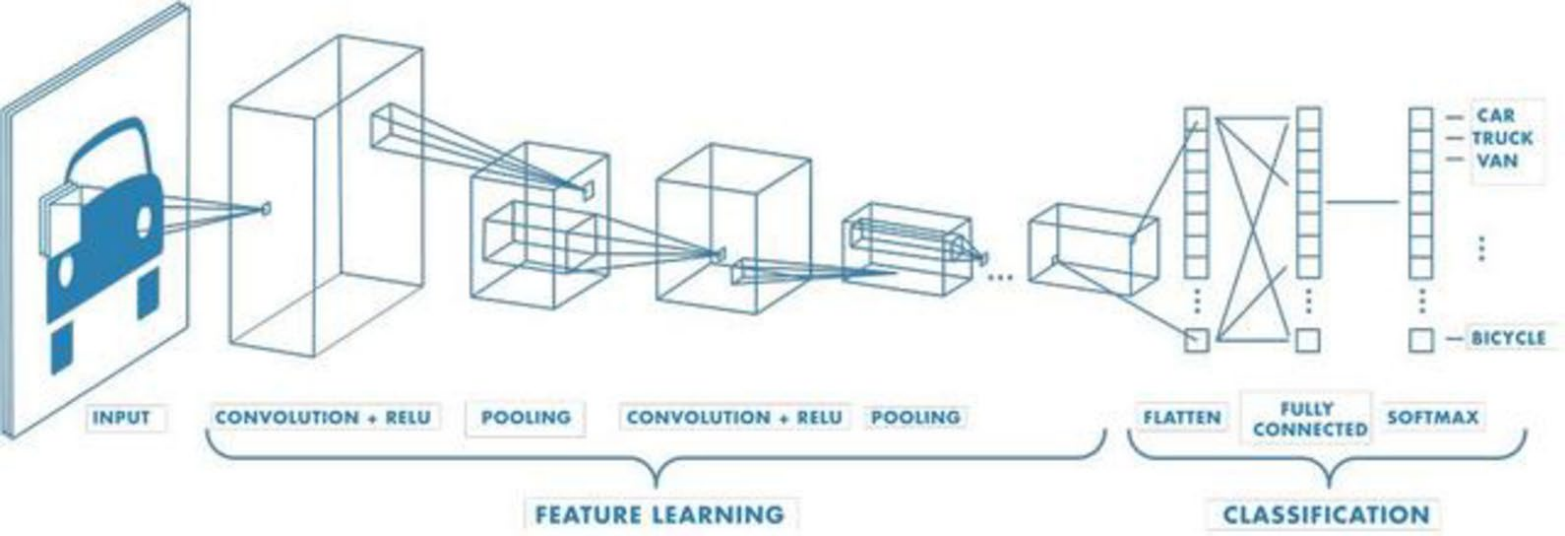
Basic Structure of CNN Network.

CNN is a type of deep neural network, whose neuron structure can adjust its weights and biases through learning. Figure 1 illustrates the basic structure of CNN. It consists of an input layer, an output layer, and several hidden layers, which often include convolutional layers, pooling layers, and fully connected layers^15,16^.

The input layer of Convolutional Neural Networks can handle multi-dimensional data. Multi-dimensional arrays are fed into the input layer of the convolutional neural network, as expressed in Eq. (1):

1 $${{\text{g}}_l}=K{{\text{g}}_{l - 1}}\left( {X,{w^{{\text{l-}}1}}} \right)$$

In the equation, *X* represents the initial input data, *w*^*1*-1^ are the learning parameters corresponding to the ${l} - {\rm 1}th$ level, denoting the bias and weights, ${{\text{g}}_{l - 1}}$ and ${{\text{g}}_l}$ represent the functions for the ${l} - {\rm 1}th$ and ${l-th}$ levels, respectively, and their output structures are both feature mapping matrices.”

The convolutional layer of a convolutional neural network is composed of feature maps obtained through convolutional operations with multiple convolutional kernels. Its essence is to perform corresponding feature extraction, as shown in Eq. (2):

2 $$X_{{\text{j}}}^{l}={\text{g}}\left( {\sum\nolimits_{i}^{M} {X_{i}^{{l - 1}} * w_{{ij}}^{l}+b_{j}^{l}} } \right),i,j=1,2, \ldots ,N$$

In the equation, $X_{{\text{j}}}^{l}$ represents the ${i-th}$ feature map of the ${{l-th}}$ layer, ${\text{g}}$ is the activation function, *M* is the number of feature maps, $*$ denotes the convolution operation, *N* is the number of convolutional kernels, $b_{j}^{l}$ is the bias of the ${{j-th}}$ feature map in the ${{l-th}}$ layer, and $w_{{ij}}^{l}$ represents the weight values of the ${t{j-th}}$ region of the ${{i-th}}$ feature map in the ${{l-th}}$ layer.

The pooling layer of CNN, also known as the downsampling layer, is located immediately after the convolutional layer. The main function of the pooling layer is to appropriately compress the model, thereby improving the robustness and computational speed of the model, and to some extent, prevent overfitting. The formula for the max pooling method is shown in Eq. (3):

3 $$p_{{\text{j}}}^{{l+1}}\left( j \right)=\mathop {\hbox{max} }\limits_{{\left( {j - 1} \right)w \leqslant t \leqslant jw}} \left\{ {q_{t}^{l}\left( t \right)} \right\}$$

In the equation, ${{w}}$ represents the receptive field, $q_{t}^{l}\left( t \right)$ denotes the input value of the ${{t-th}}$ neuron in the ${{j-th}}$ feature map of the ${{l-th}}$ layer, and $p_{{{j}}}^{{l+1}}\left( j \right)$ represents the output value of the ${{j-th}}$ feature map of the ${{l+}}1{{th}}$ layer.

After the pooling layer comes the fully connected layer. The data outputted from the pooling layer is flattened into a one-dimensional vector, which is then inputted into the fully connected layer for feature extraction. Subsequently, it is fed into the softmax classifier for classification.

#### SE attention mechanism

**Fig. 2:**
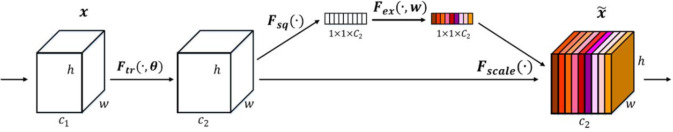
SE Channel Attention Structure.

Attention mechanisms in neural networks facilitate selective focus on specific regions of the input or allocation of varying weights to different input components, enabling the extraction of critical information from vast datasets. Among these mechanisms, Squeeze-and-Excitation Networks (SENet) represent a notable approach, introducing attention mechanisms along the channel dimension. SENet employs a secondary neural network to automatically learn the relative importance of each channel within a feature map, assigning weights that emphasize channels relevant to the current task while suppressing less informative ones. This mechanism is realized through two primary operations: Squeeze and Excitation. The Squeeze operation aggregates spatial information to generate a compact representation, while the Excitation operation uses this representation to recalibrate channel-wise feature weights. Prior to SENet processing, all feature channels are equally weighted; after SENet processing, weights vary according to channel significance, as depicted in Fig. 2. This enables enhanced focus on high-weight channels. The underlying principle involves leveraging fully connected layers to optimize feature weights via backpropagation guided by task-specific loss functions. Despite introducing additional parameters and computational overhead, SENet demonstrates substantial improvements in network learning efficacy and task performance, making it a valuable addition to attention-based architectures^16,17^.

#### Residual network (ResNet)

Convolutional neural networks (CNNs) are distinguished by their ability to automatically learn features from data, with the quality of extracted features playing a critical role in determining the accuracy of image classification tasks. Different network architectures exhibit varying capacities for feature learning, directly influencing the expressive power of the features and, consequently, the classification performance. To enhance feature quality and network effectiveness, continuous advancements in network design are essential. In deep CNNs, features are progressively refined through hierarchical integration, with greater network depth often yielding more robust and discriminative features. However, increasing network depth can lead to challenges such as overfitting and poor generalization^18,19^.

**Fig. 3:**
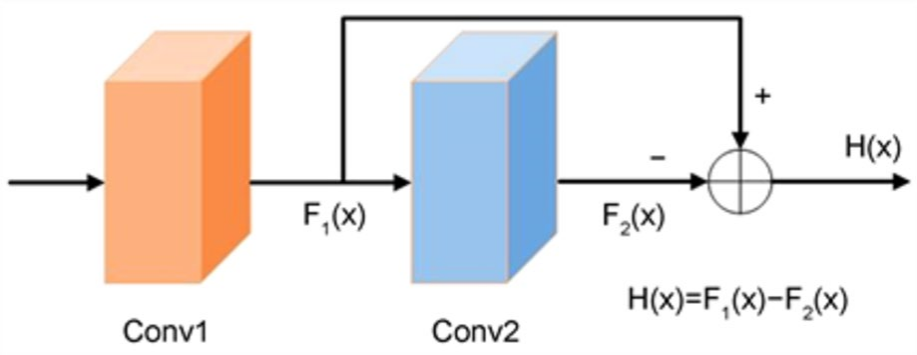
ResNet Network Architecture.

Residual Networks (ResNet) address these issues by introducing residual learning, which facilitates the training of deeper networks while mitigating performance degradation. ResNet employs a residual mapping strategy, wherein the output of two stacked convolutional layers is compared with the input to compute a residual signal. This residual serves as the learning target, simplifying optimization and enhancing gradient flow during backpropagation. The architecture of ResNet, illustrated in Fig. 3, is designed to preserve critical information while enabling efficient learning of refined features. This approach significantly improves the network’s ability to generalize and reduces overfitting, making ResNet a foundational advancement in deep learning.

4 $$H\left( X \right)={F_1}\left( X \right) - {F_2}\left( X \right)$$

The identity mapping process is illustrated in Eq. (4). Here, ${F_1}\left( X \right)$ represents the output of the shallow layer, ${F_2}\left( X \right)$ represents the output of the deep layer, and $H\left( X \right)$ denotes the residual, which tends to zero when the features represented by the shallow layer *X* are mature enough. Through the identity mapping, it is passed to the next module, effectively addressing the vanishing gradient problem. As a result, the network performance does not decrease with increasing depth.

#### Block layer

**Fig. 4:**
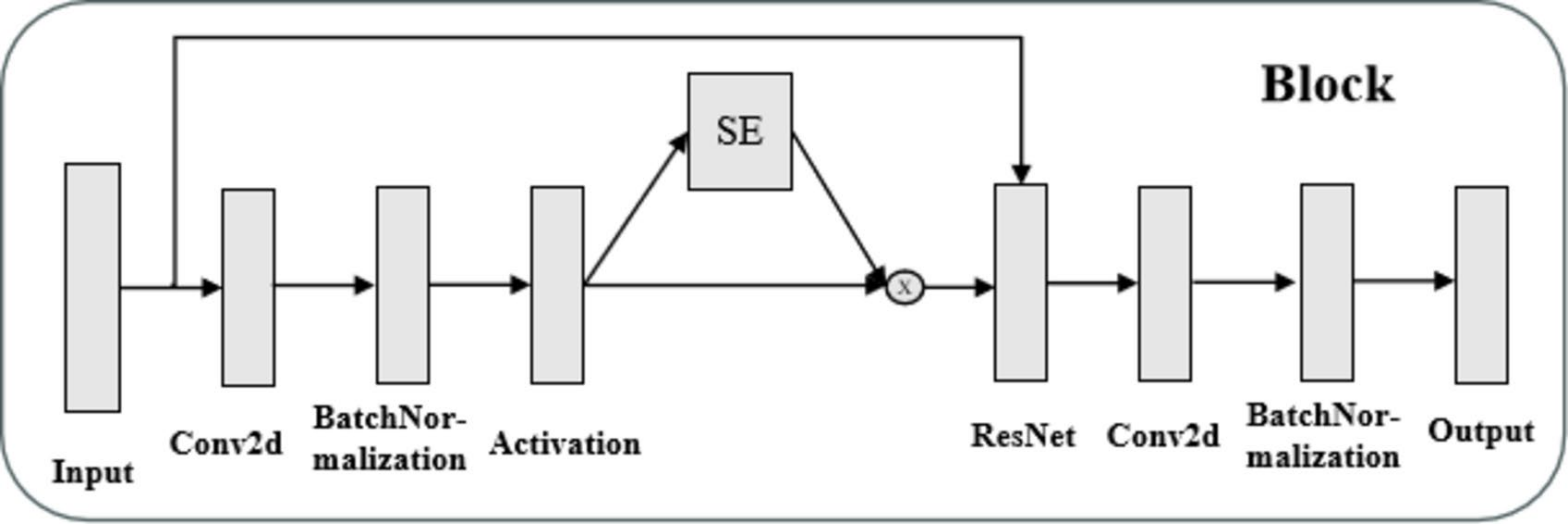
Schematic Diagram of Block Layer Network Structure.

The concept of “Block” in neural network design has emerged as a fundamental strategy to enhance computational efficiency, reduce resource consumption, and optimize network performance^20^. A Block refers to a modular computational unit that integrates multiple consecutive layers or subnetworks to perform a specific function. Drawing inspiration from modular design principles, Blocks decompose complex problems into manageable sub-problems, facilitating independent optimization. In convolutional neural networks (CNNs), a Block typically consists of various layer types, such as convolutional layers, pooling layers, and fully connected layers, connected through carefully designed patterns to achieve specific functional goals. For instance, a Block may integrate multiple convolutional layers and pooling layers to extract local features from input images or employ fully connected layers to map these features to the output space, as shown in Fig. 4. The reusable and “plug-and-play” nature of Blocks fosters flexibility in network design, enabling researchers to efficiently construct and evaluate diverse architectures tailored to specific tasks. Moreover, internal weight sharing within Blocks significantly reduces parameter counts, mitigating overfitting risks and enhancing model simplicity. This paper discusses the foundational role of Blocks in CNN architectures, their structural design principles, and their impact on model efficiency and performance.

**Fig. 5:**
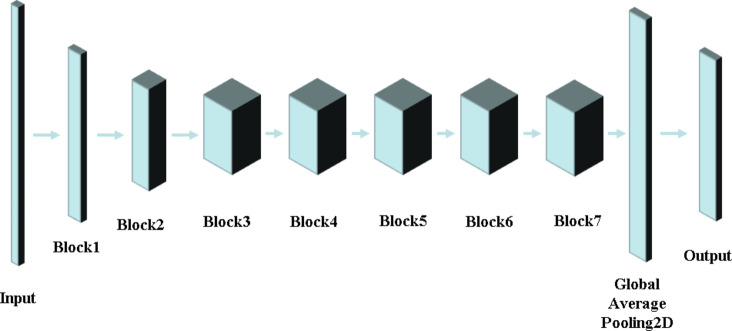
RES-SE-CNN Model Architecture Diagram.

The RES-SE-CNN model offers a significant advantage through the integration of the Squeeze-and-Excitation (SE) module, which dynamically recalibrates feature map weights by learning inter-channel relationships. This mechanism allows the model to prioritize important features, thereby enhancing its representational capacity without introducing additional network complexity. The synergy between residual connections and the SE module facilitates improved performance across multiple benchmark datasets. Although the RES-SE-CNN model has demonstrated promising results in various domains, research in its application to image recognition remains limited, and its potential in motion-based category classification via image approximation has not been explored. During training, the model is optimized using both the training and validation datasets, with continuous adjustments to its architecture based on evaluation metrics, thereby identifying the optimal configuration. The final model architecture is presented in Fig. 5, with the detailed output parameter structure provided in Table 2. To assess the model’s generalization ability, its performance is evaluated on the test set.

**Table 2:** RES-SE-CNN model architecture output parameters table.

Dense	Parameters
Input	224*224*3
Block1	115*115*16
Block2	58*58*24
Block3	29*29*40
Block4	15*15*80
Block5	15*15*112
Block6	8*8*192
Block7	8*8*320
Global_Average_Pooling2d	1280
Output	100

## Experimental setup

### Datasets

**Fig. 6:**
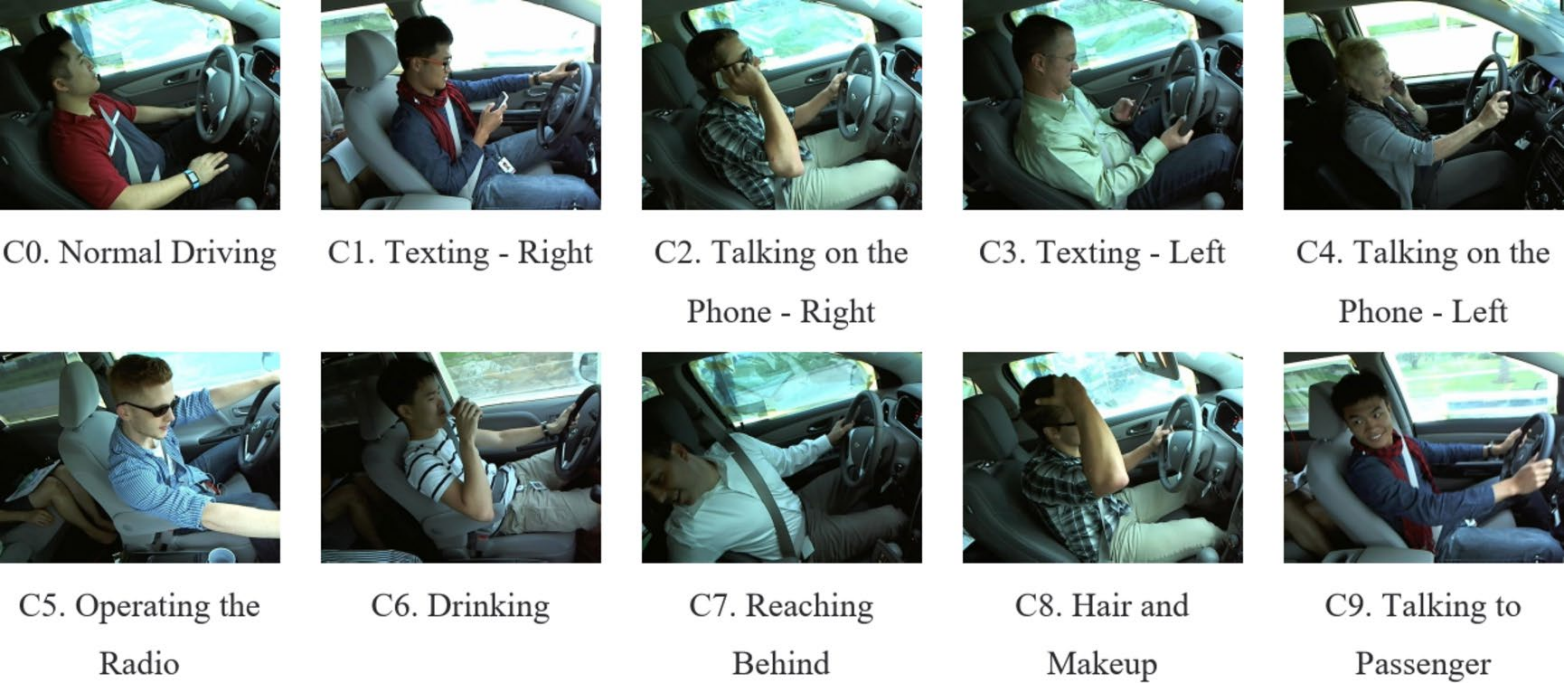
Part of the data sample diagram

**Fig. 7:**
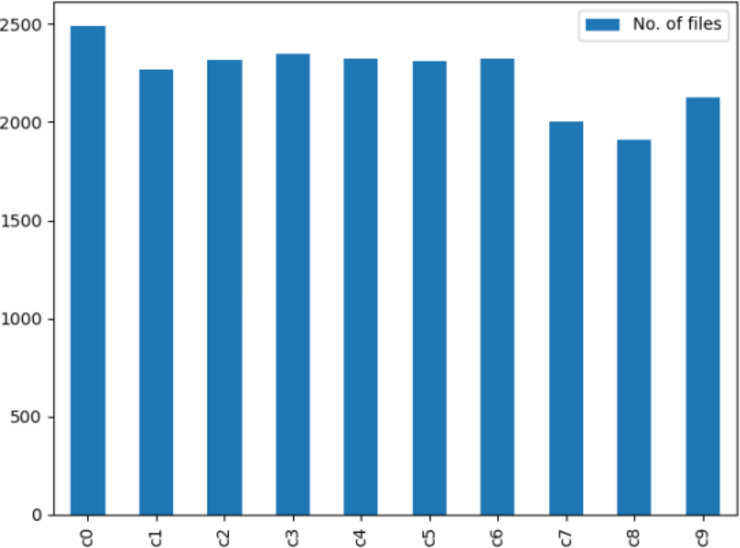
Number of data set categories.

The data used in this study is sourced from the State Farm Distracted Driver Detection dataset (https://www.kaggle.com/c/state-farm-distracted-driver-detection), initially released in 2016 and collected from vehicle cabins^21^. The competition organizer aimed to automatically identify driver distraction behaviors by analyzing images captured by dashboard cameras to address the increasing issue of traffic accidents. Figure 6 illustrates the classification of driver body postures into categories: safe (C0: Normal Driving) and unsafe (C1: Texting - Right, C2: Talking on the Phone - Right, C3: Texting - Left, C4: Talking on the Phone - Left, C5: Operating the Radio, C6: Drinking, C7: Reaching Behind, C8: Hair and Makeup, C9: Talking to Passenger), totaling ten categories. The dataset comprises 22,424 images, with the images distributed among categories as shown in Fig. 7. The data is split into training and validation sets in an 8:2 ratio.

Considering the differences in camera angles, positions, and the driver’s adjustment of the seat and steering wheel, this study aims to extract more image data from the training set to avoid unnecessary feature learning due to the excessive parameters in the neural network model, which may lead to overfitting. Deep neural networks generally require large amounts of training data to achieve optimal performance. However, in the case of limited data, data augmentation techniques, as an effective means, can expand the training dataset and improve the model’s robustness, thus effectively preventing overfitting. Currently, data augmentation is mainly applied to image data, where algorithms transform images and introduce noise to enhance data diversity.

**Fig. 8:**
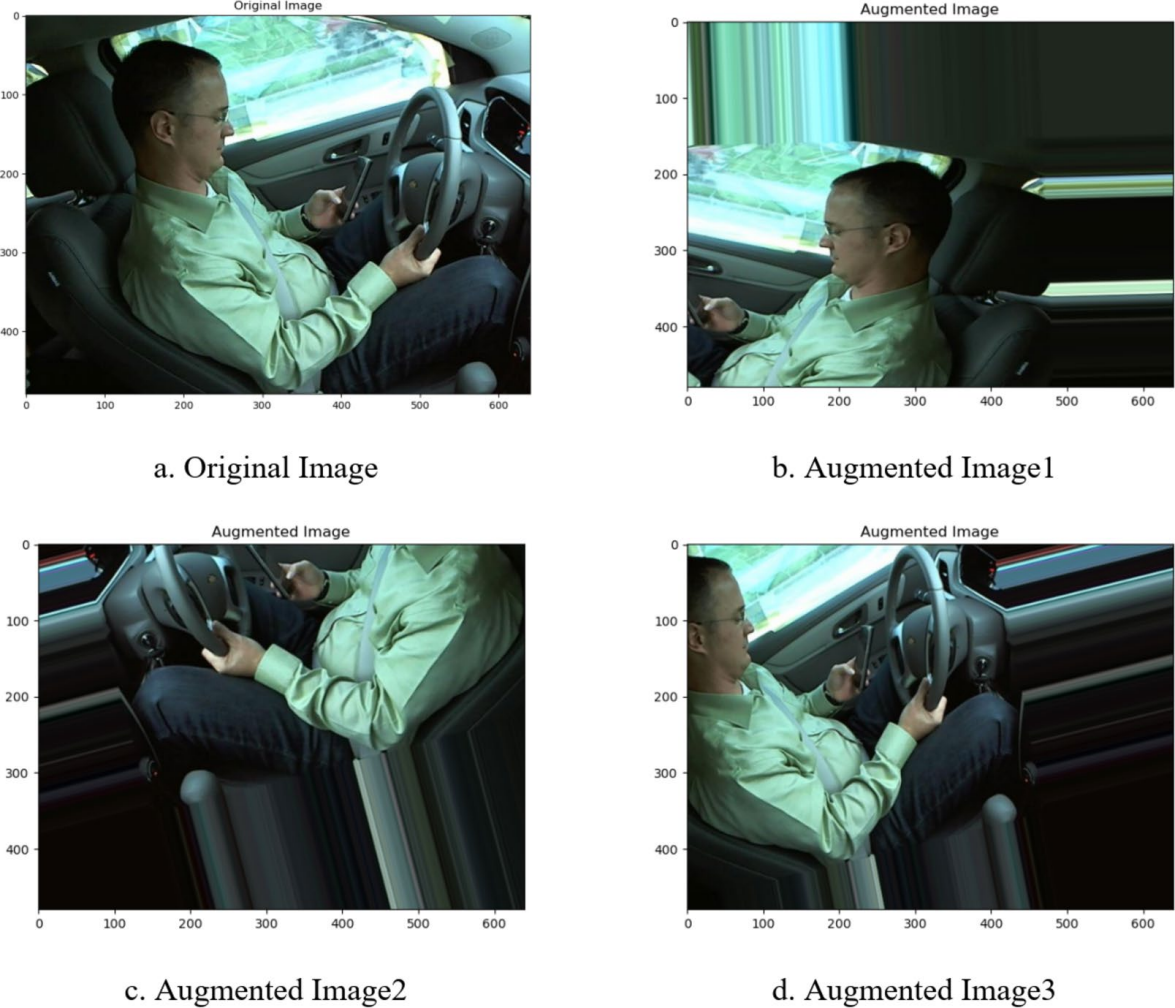
Comparison between Original Image and Augmented Images.

Image data augmentation is a technique that processes original images using various transformation operations to generate training samples with diverse features. This technique can effectively enhance the generalization ability of machine learning models and improve their performance in complex, real-world environments. Common image data augmentation methods include rotation, flipping, scaling, translation, and noise addition. Each method addresses different needs, such as variations in viewpoint, scale, position, and noise interference, helping the model better understand object features under various conditions, thus improving recognition accuracy and robustness. Specifically, the rotation operation simulates the visual effects of an object from different angles by changing the image’s direction. For example, rotating an image at different angles between 0 and 360 degrees generates multiple samples, enhancing the model’s ability to adapt to object posture changes. The flipping operation, achieved through horizontal or vertical mirroring, generates new samples, not only increasing the diversity of the dataset but also enhancing the model’s understanding of features in different object orientations. In image classification tasks, the newly generated samples through horizontal flipping help the model better recognize details on the left and right sides of objects. The scaling operation simulates the visual effects of objects at different distances by enlarging or shrinking the image, which is especially useful for object detection tasks, enabling the model to recognize objects at various scales. The translation operation generates new samples by randomly shifting the image position horizontally or vertically, simulating the visual effect of the same object at different positions, thereby improving the model’s adaptability to position variations. The noise addition operation introduces interference such as Gaussian noise or salt-and-pepper noise into the image, simulating environmental noise, and enhancing the model’s robustness to common noise disturbances. These data augmentation methods can be reasonably combined and strategically applied according to task requirements, thereby enhancing the performance of computer vision tasks. In object detection tasks, the combination of rotation, scaling, and translation can generate diverse training samples, while in image classification tasks, techniques like flipping and noise addition can improve the model’s generalization ability. In this study, images are enlarged and then subjected to angle shifts, resulting in a large number of training images. To achieve this, this paper use the ImageDataGenerator() image generator in Keras, with parameters such as vertical shift (height_shift_range = 0.5), horizontal shift (width_shift_range = 0.5), random scaling (zoom_range = 0.2), and image rotation (rotation_range = 20) to generate diversified image data. Figure 8 shows one original image and three augmented images.

### Overall workflow architecture

**Fig. 9:**
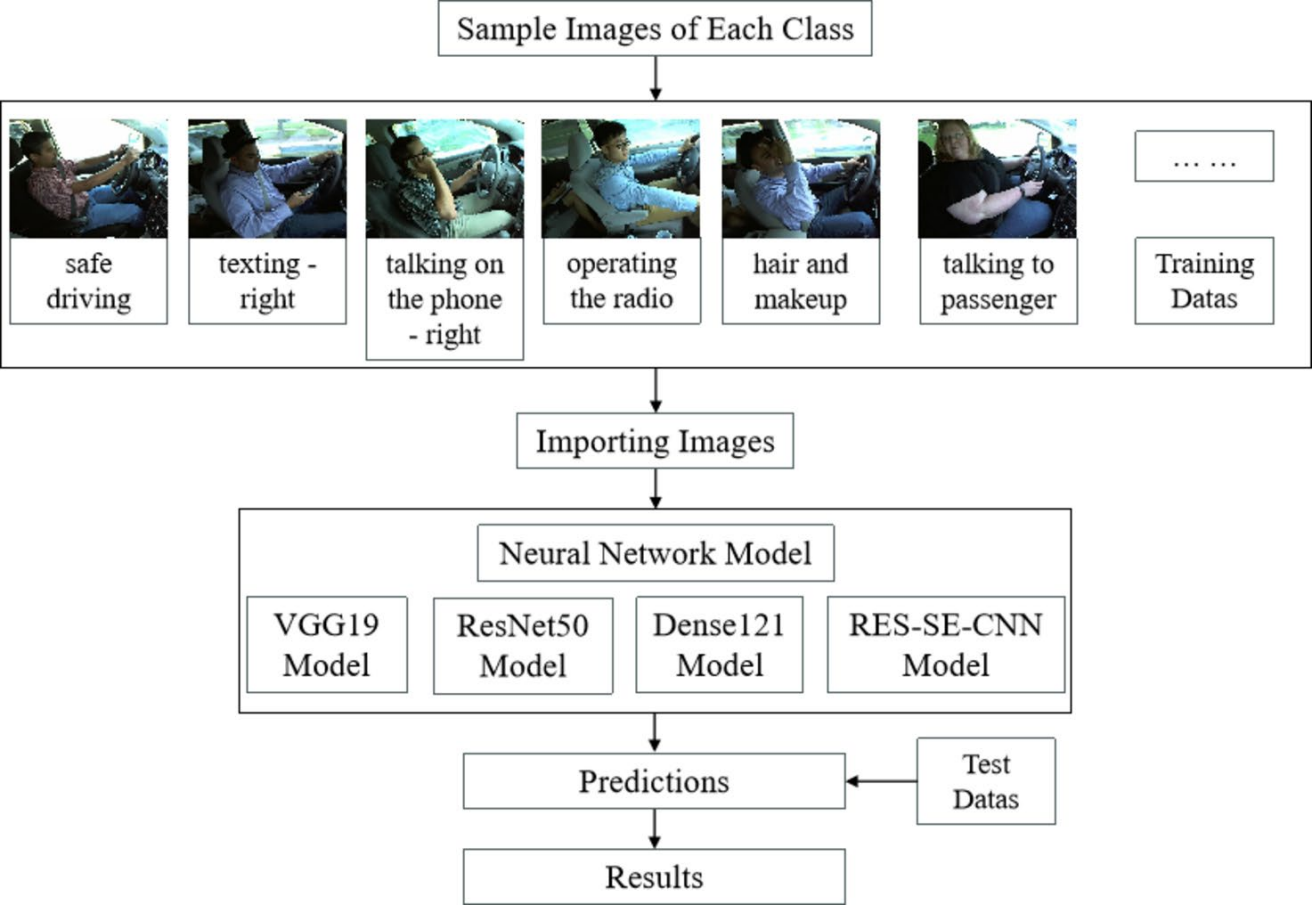
Flow diagram of distracted driving detection based on transfer learning.

The detection of distracted driving based on transfer learning includes two main steps: feature extraction and classification. The overall framework of this process is visualized in Fig. 9. First, the dataset is categorized and split into training and testing sets, with proper labeling. Next, the `ImageDataGenerator` function from the Keras module is used to batch load the data and perform data cleaning, ensuring consistent image resolution across different sizes.

Following this, models are built using four different network architectures as the foundation. Transfer learning is applied to train these models using the training and validation sets, with continuous adjustment of model weight parameters. The optimal weights are selected based on evaluation metrics. Finally, the trained models are tested on the testing set to validate their generalization capability.

### Experimental environment

The software environment for this experiment consists of the Windows 11 operating system, Anaconda3 platform, Python 3.7, and CUDA version 11.2, with TensorFlow 2.9.0 as the deep learning framework. The hardware environment includes an Intel(R) Core(TM) I7-14700KF CPU with a base frequency of 3.40 GHz and a single NVIDIA GeForce RTX 3090 GPU with 24GB of memory. The Adam optimization algorithm is used, with an initial learning rate set to 1.0e-3, a batch size of 32, and 100 epochs. The EarlyStopping function is configured with a patience value of 10, meaning that if the model’s prediction performance does not improve for 10 consecutive epochs, training will stop and the model parameters will be saved.

### Evaluation metric

This paper uses accuracy and the categorical cross-entropy loss function to evaluate the precision of the model^22^. Accuracy is the ratio of the number of correct classifications to the total number of classifications. The categorical cross-entropy loss function is used to assess the difference between the probability distribution obtained from training and the true distribution. Let the total number of samples be *n*, and the number of correctly classified samples be ${n_1}$. The formula for $accuracy$ is shown in Eq. (5), and the categorical cross-entropy loss function is given by Eq. (6) (where *c* is the total number of image categories, ${y_{i,t}}$ represents the predicted value, and ${\hat {y}_{i,t}}$represents the true value). From the formulas, the closer $accuracy$ is to 1 and the closer the ${{loss}}$ is to 0, the better the prediction performance.

5 $$accuracy=\frac{{TP+TN}}{{TP{\text{ }}+{\text{ }}TN{\text{ }}+{\text{ }}FP{\text{ }}+FN}}=\frac{{{n_1}}}{n}$$

6 $$loss =-\sum_{i=1}^n \sum_{t=1}^c y_{i,t} \: \ast \: {\rm log} \: {\hat y} _{i,t}$$

Recall is an important evaluation metric used to measure a model’s ability to correctly predict all actual positive samples^23^. Specifically, recall calculates the ratio of true positive instances predicted by the model to the total number of actual positive samples. It is based on the model’s capability to identify positive examples, providing a measure of the model’s “completeness” by determining how many of the actual positive instances were correctly found. A high recall rate indicates that the model can identify as many positive samples as possible, while a low recall rate suggests that the model may miss some positive instances. In cases where actual positive samples exist, the model’s ability to successfully recognize these positive samples is crucial. A high recall model signifies that, when actual positive instances are present, it is more reliable at predicting them as positive. In activity classification, ensuring that as many positive samples as possible are correctly identified is essential, and its definition is shown in Eq. (7). The F1 score is a metric used in statistics to evaluate the accuracy of binary (or multitask binary) classification models^24–26^. It considers both the precision and recall of the classification model, with its definition provided in Eq. (8). The F1 score can be viewed as a weighted average of precision and recall, with a maximum value of 1 and a minimum value of 0. The higher the F1 score, the better the model’s performance.

7 $${\text{re}}call=\frac{{TP}}{{TP+FN}}$$

8 $$f1 - score=2 * \frac{{precision * recall}}{{precision+recall}}$$

### Results analysis

During the learning phase, the RES - SE - CNN model likely utilizes a process similar to other deep - learning models. It begins with a large dataset of labeled images representing various driving states, including different forms of distraction. Through forward propagation, the input images are passed through a series of convolutional layers, where the model extracts hierarchical features. The unique RES - SE - CNN architecture, with its squeeze - and - excitation blocks, plays a crucial role. These blocks help in recalibrating the channel - wise feature responses, allowing the model to focus on the most discriminative features relevant to distracted driving.

In the recognition stage, the learned feature representations are then used to classify the input as a particular driving state. The model has learned the patterns and characteristics associated with distracted driving from the training data. For instance, if a driver is looking away from the road for an extended period, the model has learned to recognize the visual patterns related to this behavior in the input images. It then outputs a prediction based on the learned feature - state relationships.

The learning process was conducted using the four deep learning network architectures—VGG19, DenseNet121, ResNet50, and RES-SE-CNN models—on the dataset, and the training progress was monitored. The results are shown in Fig. 9 (the orange curve represents the training process; the blue curve represents the validation process). From the training process, it can be observed that the final loss values for the four transfer models were 0.4864, 1.1609, 0.1271, and 0.1478, respectively. The final training accuracies were 84.54%, 60.76%, 95.86%, and 95.58%, respectively.

**Fig. 10:**
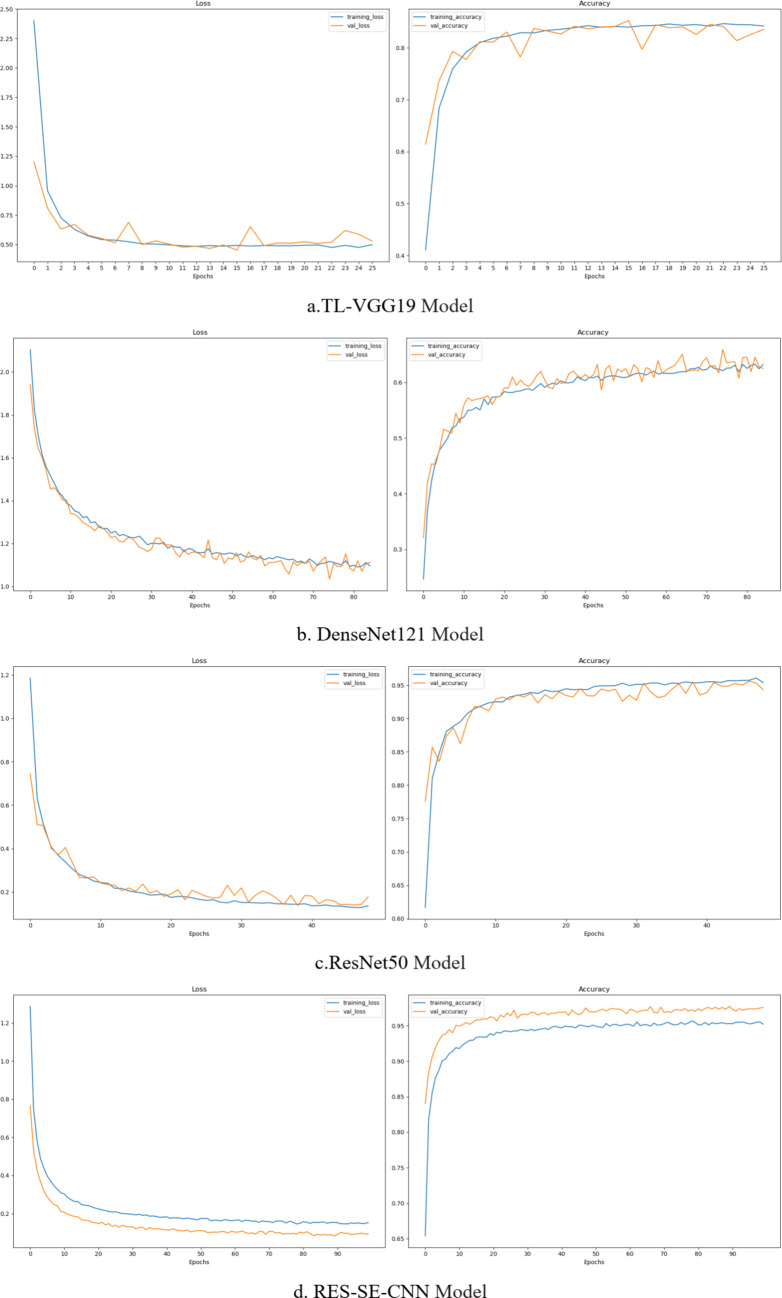
Training results of four models.

**Fig. 11:**
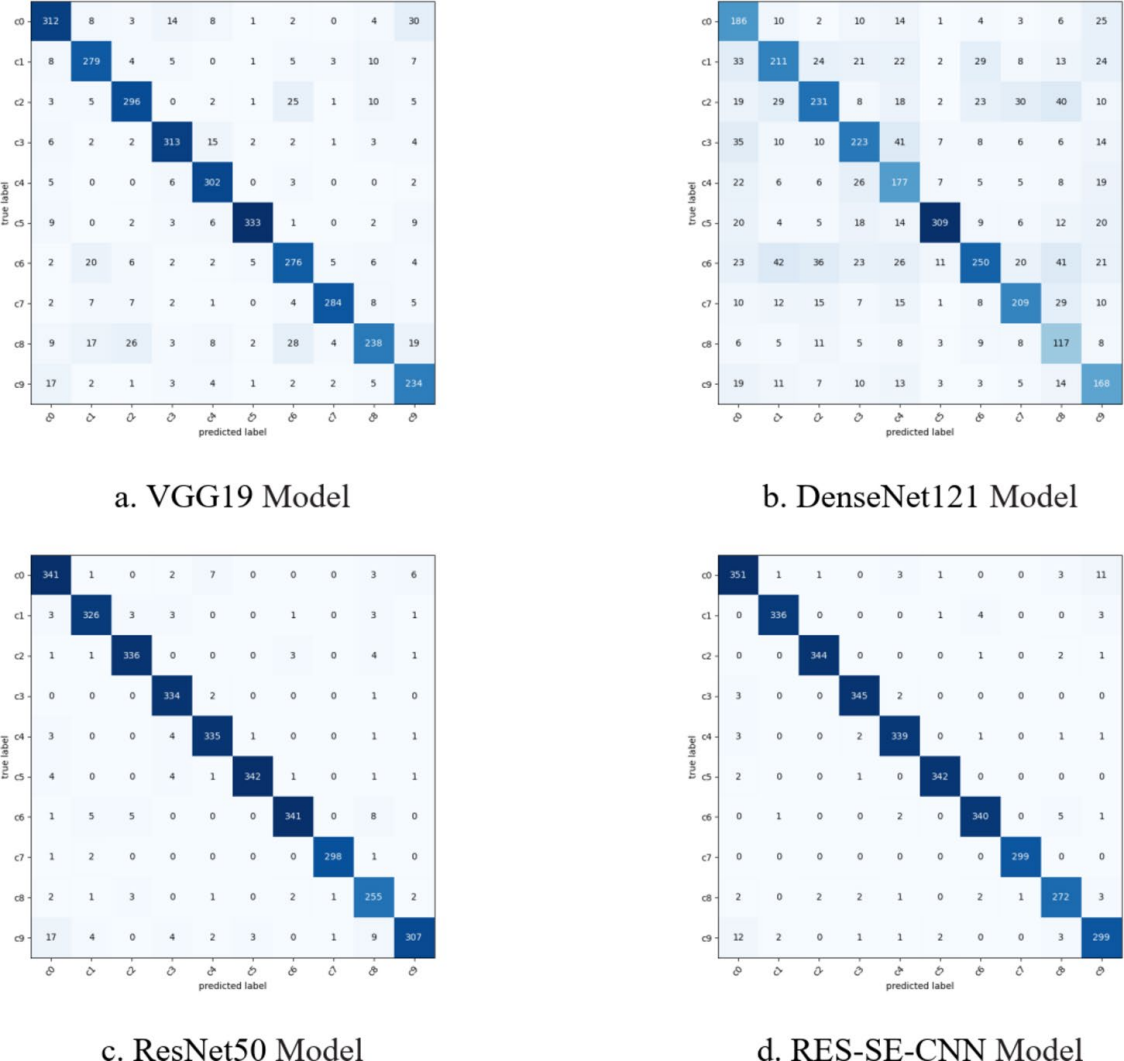
Confusion matrix diagram of four models.

**Fig. 12:**
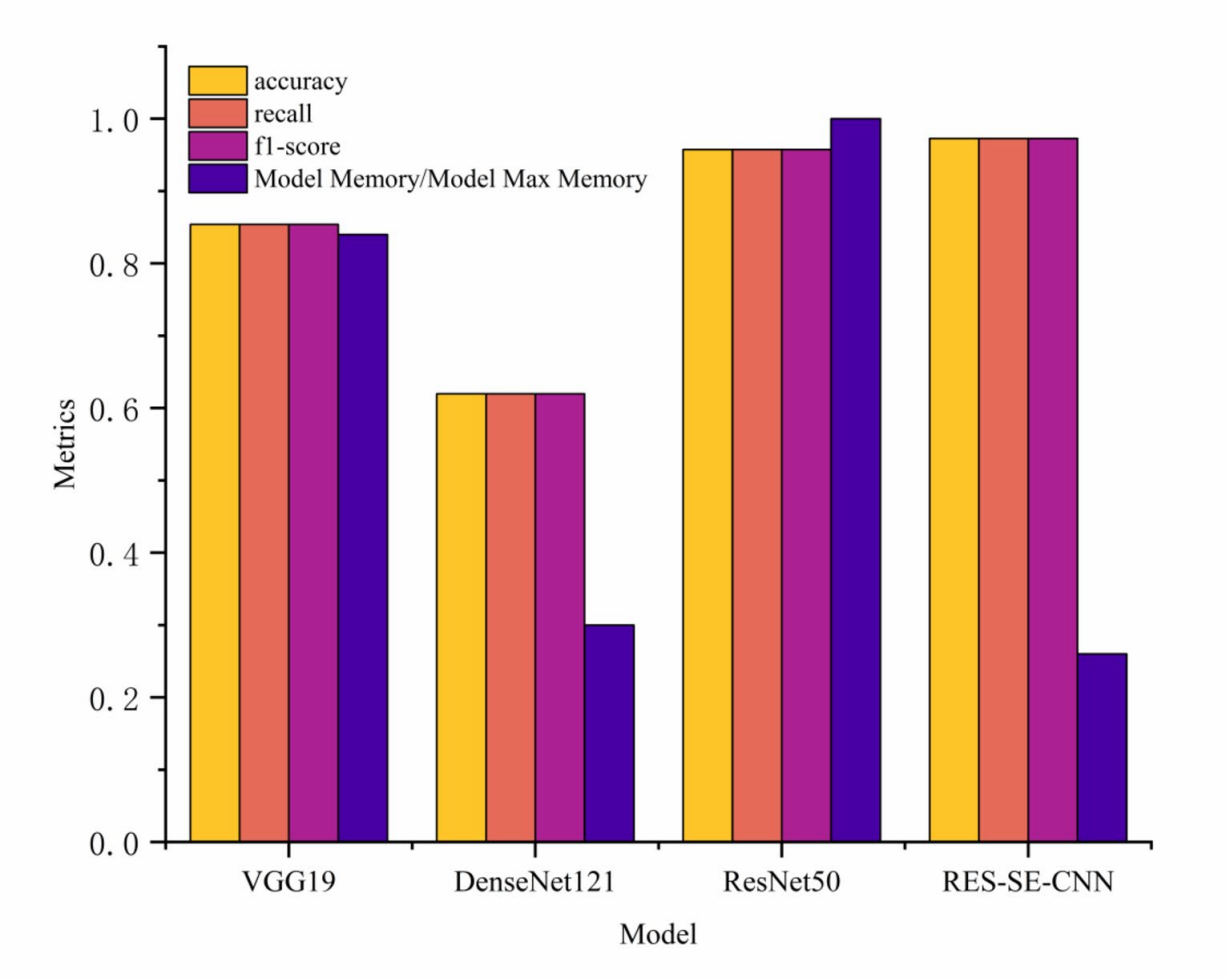
Model validation index diagram.

From Fig. 10, it can be observed that the training and validation trends of the four models generally align. Specifically, the VGG19, DenseNet121, and ResNet50 models exhibit consistent performance between training and validation phases. However, the RES-SE-CNN model demonstrates better validation performance compared to its training performance, indicating its strong generalization capability. Testing was conducted on 3,358 driver distraction state images, and the confusion matrices of the four transfer models are shown in Fig. 11 (with correct predictions along the diagonal and incorrect predictions elsewhere). The models’ accuracy, recall, F1-score, and memory usage are presented in Fig. 11 (with a maximum memory usage of 90 MB). As shown in Fig. 12, the RES-SE-CNN model achieved the highest accuracy, recall, and F1-score of 0.9729, while its memory usage was the lowest at 23 MB. The total prediction time for the 3,358 test images across all four models was 19.69 s, with a per-image prediction time of just 0.0059 s.

These results demonstrate that the intelligent driver distraction state monitoring model can accurately and efficiently assess the driver’s state, significantly enhancing driving safety.

## Conclusion

With the continuous growth in the number of motor vehicles and drivers, the road driving environment has become increasingly intricate, thereby elevating the likelihood of traffic accidents. Extensive research has indicated that distracted driving represents a significant human - related factor contributing to such accidents. Consequently, the real - time monitoring and warning of distracted driving behaviors assume critical importance. This paper puts forward an intelligent driver state monitoring approach grounded in the RES - SE - CNN model architecture. The State Farm Distracted Driver Detection dataset was adopted as the target domain for classifying and assessing ten distinct driving states. The experimental results reveal that the four models can effectively satisfy the requirements for the real - time monitoring and alerting of distracted driving. Specifically, the recognition accuracies of the four models for driver states were measured at 85.38%, 61.97%, 95.74%, and 97.29% respectively, while their memory usages were 76 MB, 27 MB, 90 MB, and 23 MB respectively. Notably, the model based on the RES - SE - CNN architecture outperformed the other three models in terms of achieving higher accuracy and lower memory consumption. This makes it a highly promising candidate for deployment within vehicle systems, thereby contributing to the enhancement of driving safety. Although the RES-SE-CNN model excels in performance and feature extraction, it has limitations such as sensitivity to multi-task learning and small sample data. In the future, these challenges can be addressed by optimizing the network architecture, reducing model parameters, integrating Transformer structures, introducing adaptive enhancement mechanisms, and improving training strategies, thereby further enhancing the model’s efficiency and generalization ability.

## Data Availability

The datasets generated during and/or analysed during the current study are available from the corresponding author on reasonable request.
